# Nurse Interns’ Experiences of Workplace Violence During Internship Programme Enrolment: A Convergent Mixed‐Method Study

**DOI:** 10.1155/jonm/7421931

**Published:** 2025-10-07

**Authors:** Khadijah Alshawush, Nutmeg Hallett, Caroline Bradbury-Jones

**Affiliations:** ^1^ Public Health Department, Faculty of Nursing, King Abdulaziz University, Jeddah, Saudi Arabia, kau.edu.sa; ^2^ Department of Nursing and Midwifery, College of Medical and Health, University of Birmingham, Edgbaston, Birmingham B15 2TT, UK, birmingham.ac.uk

**Keywords:** internship programme, nursing intern, transition, workplace violence

## Abstract

**Aim:**

The key aim of this study is to explore interns’ experiences of workplace violence (WPV) during their enrolment in internship programmes and to determine the support provided by these programmes in relation to WPV. This study will also propose improvements that can be made to ensure that internship programmes provide sufficient support.

**Background:**

Nurses, patients and organisations are adversely impacted by WPV. Nonetheless, very few studies have examined experiences of WPV among nursing interns as they transition into their working roles and enrol in their year‐long internship programmes.

**Design:**

This study employed a concurrent mixed‐methods design (cross‐sectional survey and semi‐structured interview). Data from both strands were integrated after analysis and presented in a joint display.

**Methods:**

Altogether, 143 nurse interns (123 for the quantitative phase and 20 for the qualitative phase) from two Saudi universities enrolled in a 1‐year internship took part in this study. Participants were administered quantitative surveys relating to the WPV/abuse assessment questionnaire, and interviews were held with participants who had experienced violence in the clinical workplace. Quantitative data were analysed using Statistical Package for the Social Sciences software, while thematic analysis was performed on the qualitative data.

**Results:**

The findings indicated that most nurse interns (90%) experienced violence during their internships. Moreover, it was revealed that the programme failed to adequately support them throughout their placements. A conceptual model was developed to identify the factors preventing adequate support from being provided, which made nurses more vulnerable to WPV through disempowerment.

**Conclusion:**

University, hospital and programme administrators lack coordination in providing nurse interns with WPV support. Findings offer guidance for educators, policymakers and programme developers in restructuring internship support systems.

## 1. Introduction

New graduate nurses (NGNs) (otherwise known as nurse interns) in Saudi Arabia must complete a 4‐year bachelor’s degree, followed by a year‐long nursing internship. Turnover rates increase as students transition to qualified employees, who often struggle to adapt to the new and complex work environment field [1, 2]. Furthermore, workplace violence (WPV) influences NGN turnover and burnout [3, 4]. The internship programmes in Saudi Arabia have been designed to help nurse interns transition into full‐time nurses. Nurse interns’ experiences in two large Saudi Arabian cities will be examined in this work. While the study design was primarily inductive, the findings were subsequently interpreted and contextualised through the lenses of Bronfenbrenner’s socio‐ecological model, Freire’s oppressed group theory, psychological and structural empowerment theories and transition shock theory.

### 1.1. Background

There is a shortage of nurses globally [5]. Saudi Arabia has historically faced a shortage of nurses but aims to hire 100,000 by 2030 [6]. As a result, there is a pressing need for NGNs to bridge this gap [7]. There has also been an increase in NGN turnover worldwide because of stress, job dissatisfaction and working conditions [8], as identified in two studies involving over 10000 NGNs from 50 hospitals [3, 9] and other studies of NGNs [10, 11].

The occupational safety and health administration (OSHA) has defined WPV ([12], p. 1) as:

‘Any threat or act of physical violence, harassment, or other threatening behaviour at the workplace. This can range from verbal abuse to physical assaults (and even homicide). Moreover, it can impact employees, clients, customers, and visitors’.

Meanwhile, the World Health Organization (WHO) defines WPV as physical, psychological (emotional), sexual or racial abuse [13].

In a study by Salvador et al. [14], 57.5% of participants (healthcare workers at government healthcare institutes in Abha City) stated that they had experienced WPV. Meanwhile, other studies have found that NGNs reported experiencing incivility and WPV [15, 16], as well as witnessing WPV incidents [17, 18]. In addition, many NGNs have reported experiencing WPV from colleagues (25.6%–87%) [19]. As a result, NGNs reported being continuously subjected to disempowering experiences and WPV [20].

WPV can cause emotional exhaustion, poor colleague relationships, frequent sick days and a lack of confidence among nurses [21]. According to Houck and Colbert [22], prescription errors, surgical errors, patient falls and a failure to disclose clinical concerns can occur because of workplace bullying, as WPV‐related stress and emotional fatigue can hinder clinical judgment and concentration. It can also reduce productivity and job satisfaction, cause higher absenteeism, and make nurses more likely to make mistakes [23]. Meanwhile, Johnson and Benham‐Hutchins [24] found that a hostile work atmosphere can generate high staff turnover rates, which has a detrimental effect on healthcare organisations.

To increase the retention rates of nurses, they must be educated, trained and supported. The US Institute of Medicine (IOM) recommends implementing initiatives such as nurse transition programmes to address the global shortage of nurses [25]. Compared to places like Europe and Asia, where mentorship and on‐the‐job training have taken centre stage, internship programmes received less attention [26]. Nurses in Saudi Arabia must undertake an internship in order to obtain their nursing license, which improves their clinical nursing skills, enhances their socialisation skills within the nursing role and helps them apply theoretical knowledge to clinical practice. Interns must thus complete extensive supervised training to prepare them for work as a registered nurse.

These programmes aim to make interns less vulnerable and more competent in their roles. They also increase the interns’ confidence [27] and help them overcome challenges faced during the transition period [28]. Research has proven these programmes to be effective in easing NGNs’ transition to practice [29] and thereby reducing turnover while enhancing their clinical skills [30]. Nonetheless, little research has examined whether such programmes help interns deal with WPV, particularly in Saudi Arabia [26].

Several descriptive studies have examined the characteristics, magnitude and consequences of WPV for individuals and healthcare organisations [31, 32], and some have examined staff violence between employees [33, 34] and patients [35, 36]. In Saudi Arabia, nurses and other healthcare professionals experience different acts of WPV, including physical violence, verbal violence, and bullying [14, 37–40]. However, these studies include Saudi and non‐Saudi healthcare professionals but not interns. No existing studies have explored WPV relating to internship programmes, particularly in the context of Saudi Arabia. Data indicate that WPV is a significant issue facing nursing interns. It is thus vital to understand how internship programmes can overcome this problem. Therefore, the present study investigates the WPV experiences of nursing interns throughout the internship programmes and the levels of support that such programmes provide to nursing interns.

This study aimed to address the research questions and objectives presented below:

1. Investigate how nurse interns experience WPV during nurse internship programmes.
  a. Explore the frequency of WPV incidents experienced by nurse interns (quantitative).
  b. Identify the types of WPV experienced by nurse interns (quantitative).
  c. Understand the impact of WPV on nurse interns (quantitative + qualitative).
2. Investigate the extent to which nurse internship programmes support nurse interns during the experience of WPV.
  a. Explore how much nurse interns know about reporting WPV incidents (quantitative + qualitative).
  b. Explore the degree/type of support nurse internship programmes provide to interns regarding WPV (qualitative).
  c. Understand the factors (if any) that hinder the nurse internship programmes’ support of WPV (qualitative).
3. Understand how nurse internship programmes might be improved (if necessary) to support nurse interns in dealing with WPV.
  a. Identify the needs of nurse interns from nurse internship programmes when dealing with WPV (qualitative).
  b. Suggest how nurse internship programmes might be improved (if necessary) to support nurse interns in dealing with WPV from the nurse interns’ perspectives (quantitative + qualitative).

## 2. Methods

### 2.1. Design

This study was guided by a pragmatic approach [41], and the research design employed was a convergent mixed‐method design to thoroughly address the research question, ensuring comprehensive coverage of both statistical trends and contextual depth [42]. A convergent design was used where both methods were given equal priority and conducted simultaneously, allowing an in‐depth comparative analysis to synthesise diverse results.

**Figure 1:**
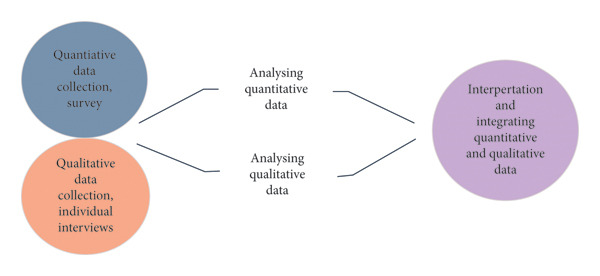
A process of the convergent mixed‐method study.

A cross‐sectional survey and qualitative interviews with nurse interns were conducted to better understand their WPV experiences during their programmes. Data were collected and analysed simultaneously, while data were integrated during the inference stage (see Figure 1). Integration occurred at multiple levels: at study design (convergent mixed‐methods design), at methods (connecting and merging data) and at interpretation and reporting (narrative and joint display). A pragmatic approach was employed to evaluate the completeness of the data, and the quantitative and qualitative findings were combined to provide deeper insights that could not be achieved using one method alone.

### 2.2. Setting and Sample

This study was conducted at two large city universities in Saudi Arabia that developed guidelines for the internship programmes provided by many national hospitals. The placements include rotations in different speciality areas (i.e., emergency, medical, surgical and intensive care units). To be included in the study, nurse interns had to be Saudi nationals who had already graduated from the bachelor nursing programme and were currently undertaking the internship programmes. The eligible participants (*n* = 200) were asked to partake in both research phases (anonymised survey and semi‐structured interviews). Data were collected between August 2021 and January 2022.

### 2.3. Data Collection

Nonprobability purposive sampling was employed for the online anonymised survey, and the Raosoft calculator was used to determine the optimal sample size with a 95% confidence level, 5% margin of error, and 5% response distribution. Consequently, a sample of 143 interns was considered suitable from a standard cohort of 200 interns representing the overall cohort from the two universities. WhatsApp was used to contact potential participants, while programme coordinators emailed them the recruitment materials and online survey links. Participants were asked to provide contact details if they wanted to participate in the qualitative study (interview). A pilot study was conducted with 13 nurses to check the questions’ reliability and internal consistency.

The questions were designed to uncover demographic and work‐related information (i.e., gender, age, university, marital status, specialities, and programmes enrolment duration). Participants were asked to complete the WPV/Abuse Assessment Questionnaire (WPVAQ) [43] so that researchers could understand their perceptions of WPV. Two studies modified the WPVAQ to suit the Saudi Arabian context, Aljanabi et al. [44], Alkorashy and Al Moalad [45], retaining English language while adapting items to local cultural nuances, such as replacing references to nursing unions with the Ministry of Health’s emergency call centre number. The adapted WPVAQ was found to have high reliability (Cronbach’s alpha of 0.977 and 0.76 in two studies) and validity [44, 45]. Cronbach’s alpha was found to be 0.958 in this study. The questionnaire contains four sections and covers WPV incidents, reporting, consequences, solutions, nurses’ knowledge and legal rights.

Participants (*n* = 12) who provided details in the questionnaire and reported experiencing or witnessing WPV in the workplace were asked to participate in the semi‐structured interviews (purposive sampling (*n* = 12) followed by snowballing (*n* = 8) to include hard‐to‐reach perspectives). Selecting participants from a diverse range of clinical specialities (in different hospitals), geographic locations (Riyadh and Jeddah) and with equal gender representation enriched the diversity and representativeness of the sample. The findings could then reflect the heterogeneity of the population of interns, facilitating a more rounded understanding of their experiences. Because of the COVID‐19 restrictions, all participants responded to the interviews via Zoom. Audio recordings of the interviews were made and then professionally transcribed. The same prompt questions were used in all interviews, each lasting 45–60 min. The interviews (*n* = 20) were carried out in English. Interviews and coding were conducted independently by K.A. and reviewed by N.H. and C.B‐J. Following the 15th interview, no new themes emerged, and subsequent interviews reflected the same patterns and themes, indicating that the data saturation point had been reached. However, considering the diversity of the interns’ rotation through clinical specialities during the programmes, an additional five participants were interviewed to strengthen the conclusion.

### 2.4. Data Analysis

The prevalence of WPV was analysed using the Statistical Package for the Social Sciences (SPSS) version 20. Furthermore, a binary logistic regression analysis was used to predict exposure to violence in the workplace through demographic predictors. The interviews were audio recorded, transcribed verbatim and coded using NVivo. An inductive approach was then employed to perform thematic analysis. The interview guide served as a checklist tool, ensuring that important topics related to the problem were covered while maintaining a structured approach to the interview. Braun’s [46] six steps of the thematic analysis process were utilised: data familiarisation, code creation, theme construction, theme review, theme significance determination and reporting of findings.

The good reporting of a mixed‐methods study (GRAMMS) guidelines were followed to ensure that the findings were explicit and comprehensive [47] (see Supporting file 1). GRAMMS was also employed to improve the rigour and credibility of the mixed‐method study.

### 2.5. Ethical Considerations

The ethics committee at the two universities provided ethical approval for the study to proceed (ref: 2f.70 &21/0546; date 28/02/2021), and the study complied with the Declaration of Helsinki. Participants provided written consent after receiving written explanations of the study’s purpose, risks and benefits. If participants required any clarification, they were urged to contact the researcher. They were also informed of their right not to participate, but they could not withdraw after completing the online survey because of the anonymity of the process. However, participants were informed that they could withdraw at any point during the survey by closing the browser without submitting, with no data being recorded. The initial survey was designed to be fully anonymous. No identifying information (IP addresses, emails, etc.) was collected within the survey platform itself. In case of any negative thoughts or feelings after the survey, the contact details of university programme coordinators for interns were provided, and participants were assured that referrals were optional and would not affect their internship evaluations. Interviewees were informed that the study was confidential, and their contributions would remain anonymous. They had the right to withdraw at any point, even after the interview had begun.

## 3. Findings

### 3.1. Participant Characteristics

Of the 200 surveys distributed, 132 (66%) were completed, of which 123 (61.5%) were considered valid. Most participants came from one university (62.6%), were male (61%), were less than 25 years of age (92.7%) and had been enrolled in the programmes for more than nine months (65%) (see Table 1).

**Table 1:** Frequency and percentage distribution of interns according to socio‐demographic and work‐related characteristics.

Variables	Groups	Frequency (%) *n* = 123
Gender	Male	75 (61%)
	Female	48 (39%)

Age	Less than 25 years	114 (92.7%)
	25–35 years	9 (7.32%)

Months of programme enrolment	0–4 months	30 (24.4%)
	5–8 months	13 (10.6%)
	9–12 months	80 (65%)

University	Jeddah	46 (37.4%)
	Riyadh	77 (62.6%)

Marital status	Single	112 (91.1%)
	Married	11 (8.9%)

Speciality area	Male ward	27 (22%)
	Female ward	13 (10.6%)
	Emergency department	48 (39%)
	Patient clinics	6 (4.9%)
	Other (NICU, all departments, ICU, surgical ward and medical ward)	29 (23.6%)

Moreover, the interview participants included 20 Saudi nursing interns (50% female, aged 22–24 years) from two universities, all enrolled in hospital internship programmes (3–12 months duration). Participants represented eight hospitals and nine speciality areas, with most (55%) in their final programme month.

### 3.2. Survey Findings

Altogether (*n* = 123) 90.2% of interns reported experiencing or witnessing WPV during their internship programmes, and most interns reported WPV taking place during day shifts (48%) (shown in Table 2).

**Table 2:** Frequency and percentage distribution of interns’ experience of the incidence of workplace violence/abuse, the effect of violence in the workplace by interns, as well as reporting and management responses.

		Frequency (%) *n* = 123
Have you ever experienced or witnessed workplace violence?	Yes	111 (90.2%)
	No	12 (9.8%)

The shift you were working when the most severe/violent incident took place	Day	59 (48%)
	Evening	6 (5%)
	Night	18 (14.6%)
	Weekend	16 (14%)
	Holiday	17 (15%)
	Other (at any time and any day)	2 (2%)

Did you continue working after the incident?	Yes, I continued working	52 (42.3%)
	No, I refused to continue working	42 (34.1%)
	No, I was sent home	17 (13.8%)

Effect of violence on work performance	Difficulty concentrating on the job	29 (26.1%)
	No effect	28 (25.2%)
	Difficulty working in an environment that reminds me of past incidents	17 (15.3%)
	Physical symptoms, such as headaches and stomach aches	10 (9.0%)
	Psychological symptoms, such as fear	10 (9.0%)
	Hypervigilance, easily startled	9 (8.1%)
	Not fearful, but physical injuries have decreased my ability to work	6 (5.4%)
	Other	2 (1.8%)

Transferred to a new unit or worksite because of feeling unsafe related to a violence/abuse	Yes	15 (13.5%)
	No	95 (85.5%)
	Other	1 (0.9%)

Do you know how and where to report incidents of violence in your work setting?	Yes	110 (89.4%)
	No	13 (10.6%)

Reporting the violence to the management	I Reported all incidents to the management	63 (56.8%)
	I Reported some incidents	38 (34.2%)
	I did not report any incident	10 (9%)

Who else have you reported incidents of violence to, if any?	Police or district attorney	22 (17.9%)
	Lawyer	20 (16.3%)
	937 Centre	8 (6.5%)
	None	27 (22%)

Management response towards the violent attack	Management was supportive and tried to find solutions	48 (48.0%)
	Management was supportive but nothing was done to solve the problem	28 (28.0%)
	Management was neither supportive nor blaming	13 (13.0%)
	Management intimidated or discouraged me from reporting incidents	11 (11.0%)
	Management harassed or blamed me when I reported incidents	11 (11.0%)

Providing relief for victim nurse so that he/she could leave after the incident	Yes	66 (66.0%)
	No	45 (45.0%)

How concerned are your mangers about your safety at work?	Not very concerned	88 (71.5%)
	Somewhat concerned	20 (16.3%)
	Very concerned	15 (12.2%)

What degree of control do you feel you have over your safety in your workplace?	No control	58 (47.2%)
	Some control	44 (35.8%)
	A lot of control	21 (17.1%)

**Figure 2:**
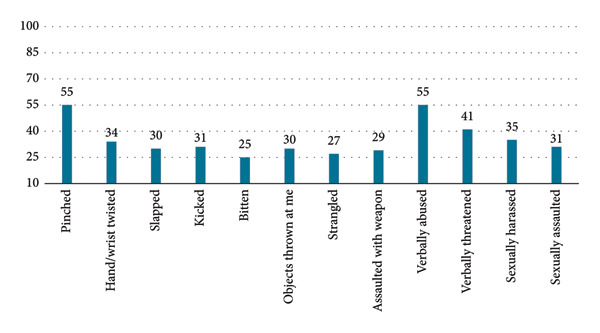
The total number of each type of workplace violence incident.

The frequency of WPV varied from one to three times to nine times or more. With a mean score of 0.640, verbal abuse was the most commonly reported form of violence experienced by interns. After that, there were reports of being kicked (mean score of 0.405) and pinched (mean score of 0.441). Strangulation had the lowest reported frequency (mean score: 0.189) (See Figure 2) of all violent incidents.

**Figure 3:**
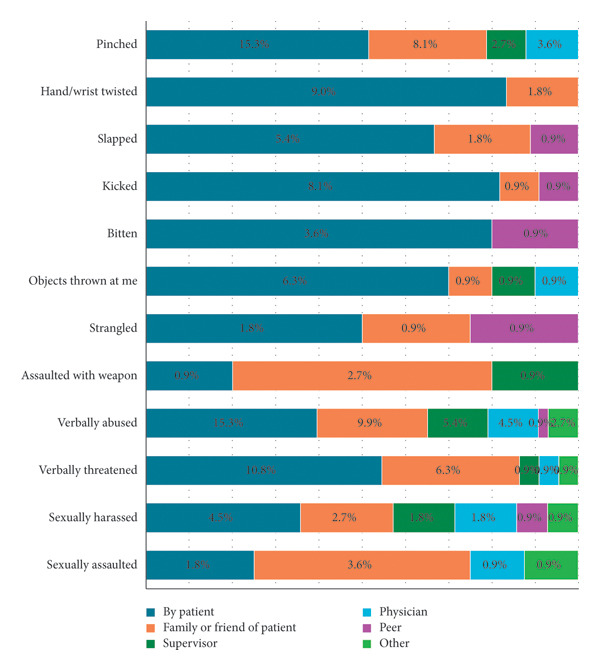
Percentage of nurses’ responses to the source of occurrence of types of violence/abuse in the workplace.

Even though the most common sources of WPV were patients and their families, reports were also made of verbal assaults and threats on the part of supervisors (5.4%). Weapon assaults by patients, relatives and supervisors were also reported (0.9%, 2.7% and 0.9%, respectively). In addition, reports were made of sexual harassment and assault by patients (4.5%), patients’ family members (2.7%), supervisors (1.8%), physicians (1.8%) and peers (0.9%) (See Figure 3).

The findings revealed that 52% of interns continued to work after experiencing a WPV incident, while 34.1% did not (see Table 2). Moreover, 26.1% had difficulty concentrating on their work because of WPV, and psychological symptoms were reported by 9.0%. Finally, 9.0% reported experiencing physical injuries. Only 56.8% reported incidents, even though most interns knew how to report them. In general, management teams were reported to respond supportively (48.0%). However, 28.0% reported that insufficient (if any) action was taken, with most interns (71.5%) believing that the manager’s concern for their safety was inadequate, and the majority of interns (85.5%) were not transferred to any new ward after the incidents. Most interns (47.2) thought they had no control over their safety. Furthermore, although 85.4% of participants received training on WPV prevention, almost half reported that it was policy‐focused. More than half of the participants attended self‐defence courses, and 69% knew their legal rights. Finally, 58.5% of participants thought that training could be a solution to improve working conditions.

Table 3 presents the results of the binary logistic regression analysis on the relationship between demographic variables and WPV incidents. The dependent variable was the interns’ exposure to WPV (1 = yes, 0 = no), while the independent variables were age, gender, marital status, speciality area, university and length of the programmes’ enrolment. There was a notable relationship between WPV and age (odds ratio of 0.149, *p* = 0.017), with findings indicating that younger nurses (the younger group are the reference) are more likely to experience or witness WPV than older nurses and that the highest likelihood of experiencing WPV was in the emergency department (*p*‐value of 0.018, OR of 1.593) (male ward was the reference). However, no significant results were identified in other speciality areas. This finding suggests that violence risks increase as individuals progress beyond the early stages of their enrolment in the programmes.

**Table 3:** Binary logistic regression between demographic variables and a nurse’s exposure to or witnessing violence in the workplace (*n* = 123).

Predictors	OR	*p*‐value	C1 95%
Gender	1.350	0.753	[0.220, 8.278]
Male (reference)			
Female			

Age	0.149	0.017	[0.033, 0.677]
Less than 25 (reference)			
More than 25			

Marital status	4.468	0.160	[0.559, 35.730]
Single (reference)			
Married			

Speciality area			
Male ward (reference)			
Female ward	1.310	0.885	[0.030, 56.526]
Emergency department	1.593	0.018	[1.085, 2.339]
Patient clinics	3.970	0.398	[0.160, 98.100]

University	0.386	0.291	[0.066, 2.265]
University A (reference)			
University B			

### 3.3. Interview Findings

Five themes were identified in the interviews, all of which capture the widespread experience of WPV in clinical settings throughout nursing internships and the support given to nurses by programme organisers. These themes were uncovering the reality and advantages of the internship programme, factors impeding programmes support, navigating the dual effects of WPV and insufficient support for interns, and nurse interns’ needs and recommendations for improving working conditions. All themes were grounded in data from both universities, and then the manifestation of these themes was specifically compared between the two groups, ensuring no substantive regional differences in core experiences.

Normalisation was evident in all the themes, with interns and most of their preceptors acknowledging WPV as a necessary component of the work. Even after WPV was reported, no action was taken. Verbal abuse and bullying were admitted as an essential step in the nursing transition process.

‘Unfortunately, this is a common occurrence among my hospital colleagues, who believed that disrespect and having a nurse act as though you are under command whenever they speak to you is the norm’ (Participant 20).

#### 3.3.1. Unveiling the Reality

The first theme focused on answering the following research question: How much WPV do nursing interns experience while enrolled in the programmes? Interns experience many WPV incidents during their placements. All the interns who participated in the interviews had either experienced or observed WPV. One intern reported that they were *‘the weakest group, the group that is most commonly abused and subjected to violence’ (Participant 13).*

Moreover, participants reported that patients could be physically violent towards interns, but staff and physicians verbally abused them the most. One employee also reported sexual harassment. In addition, the key causes of WPV were found to be patients themselves and a poor understanding of the work environment reality. Some interns reported that they were not afforded any discretion regarding mental health issues or aggressive patients by their preceptors. Interestingly, male interns reported all incidents of physical violence, but only female interns reported verbal abuse and sexual harassment.

#### 3.3.2. Advantages of the Internship Programmes

This theme relates to the second research question: How do nurse internship programmes support nurse interns during the WPV experience? Even though interns reported being exposed to WPV, they agreed that the programmes should support them and enhance their resilience. This theme outlines the programmes’ advantages.

‘This internship has taught me that the experiences encountered during this period, whether good or bad, you won’t feel lost’ (Participant 8).

Participating in the programmes can make transitioning from intern to qualified nurse easier because it equips nurses with the experience needed to be safe patient carers. A number of interns reported that their preceptors and programme coordinators supported them during WPV incidents. Nonetheless, most of the proposed solutions involved switching patients or providing emotional support to interns: *‘The preceptor just distanced me from [the patient] and said that she would go [to the patient] instead’ (Participant 13).*

#### 3.3.3. Factors Hindering Internship Programmes Support

This theme relates to the perceived support received by the nursing interns, who believe that certain vulnerabilities impact the effectiveness of the programmes’ support and increase the risk of WPV. These factors can be divided into programmes, hospitals and universities.

##### 3.3.3.1. University Factors

The university developed the programmes’ guidelines, which hospitals used to prepare interns for the programmes. The university’s proactive preparation, reactive support and follow‐ups can influence the reporting of violence and protect interns in the future. One participant stated, ‘*They could provide more education about WPV when we enter the hospital for the first time alone’ (Participant 6).*

The university is responsible for the interns, even though they are being trained at the hospital.

‘It would help us to manage difficult WPV situations if they were to check on us or contact our coordinator frequently’ (Participant 5).

##### 3.3.3.2. Hospitals Factors

Work overload is also a significant issue that can contribute to WPV. The interns highlighted a culture shock here, stating that everything was new to them, and the staff were often too busy to help them. ‘W*hen we went to the hospital, it was a cultural shock because there was a massive difference between the two settings’ (Participant 20).*

Moreover, the interns reported experiencing ignorance, gossiping and abuse from nurses, as well as poor support from their preceptors regarding WPV: ‘*We had a problem with preceptors because they were not supportive of the students’ (Participant 12).*

There are three potential reasons for the attitudes demonstrated by the staff and preceptors:

a. Lack of confidence in the interns’ skills, as some preceptors consider them insufficiently qualified students to deal with patients: ‘*I do not know if it is about their ego or a lack of confidence in our abilities’ (Participant 1).*
b. Saudisation, a project initiated by the Saudi government to replace non‐Saudi nurses with Saudi nurses:

‘They say you are Saudi; you’ll take our place. The Saudisation has made them feel I am there, so they don’t want to teach me that much because they feel we will take their place’ (Participant 10).

c. Poor support from managers: ‘If the unit managers checked on the staff, they would notice these things (i.e., abuse)’ (Participant 2).

##### 3.3.3.3. Internship Programmes Factors

The participants also highlighted a number of programmes‐related factors that are believed to impact WPV, including poor communication between the programme organisers and between the coordinators and interns and a belief that the programmes need to support and train interns adequately.

‘They [the university programme coordinators] do not ask us about the programme or our progress. They just know we are there [in the hospital]’ (Participant 16).

A lack of communication was identified between programme coordinators and between programme coordinators and interns. It is thus expected that reporting WPV would be challenging.

‘Even though we all experience WPV, nobody reports it. . Finding them [the programme coordinators] is too difficult, and some are never available’ (Participant 8).

Most interns who had experienced WPV were unhappy with the support they received from programme coordinators. For instance, participant 2 stated, *‘I made a report once to the programme coordinator about the attitude of my preceptor, and she simply assigned me a different preceptor.’*

There was a consensus among participants that insufficient education regarding WPV contributed to WPV because more education would enhance their knowledge of the issue and how to manage it.

‘Education about WPV is the most significant thing that is lacking. My internship only focused on work and skills, with no education about WPV’ (Participant 10).

#### 3.3.4. Navigating the Dual Impacts of WPV and Insufficient Programme Support

The participants agreed that WPV emotionally impacted the interns because of their lack of support. These impacts include stress, depression, fear, hopelessness and anger. In line with this, Participant 9 stated, ‘*I hated it. I cried a lot that day and needed more than the psychological support from the programme.*’

Participants also highlighted a number of professional impacts of WPV, such as higher intentions to leave the role because of insufficient support received. Many felt that they did not want to continue in the programmes or work for the same hospital in future.

‘I feel very depressed, and crying is now a daily habit. I feel like sleeping most of the time, and I lose focus. I feel like I am destroying myself just to finish this year and want to drop out of the programme’ (Participant 19).

Another professional impact highlighted by the participants was confusion about the role of the interns and student nurses, which was believed to result from the poor communication between universities and hospitals*: ‘The way they talk with you is aggressive because you are still a student. But I am not a student; I am an intern’ (Participant 3).*

A number of interns feared that reporting WPV would impact their careers and that they would not be able to work in the same hospital after the internship. For instance, participant *6 stated that they feared punishment: ‘I feel like if I report on someone, I will be punished.’*

#### 3.3.5. Nurse Interns’ Needs and Suggestions for Improvements to the Programmes and Work Conditions

This theme addresses the final research question (i.e., how can the nurse internship programmes be improved to better support nurse interns in dealing with WPV?). The participants highlighted their needs related to WPV and made recommendations for improvements that could be made to help them cope with WPV and improve their work conditions during the period of transition. More specifically, they highlighted their need for education, support and effective communication. The interns reported that WPV is likely inevitable: *‘WPV can happen anywhere, but we just need to know how to deal with it’ (Participant 8).*

## 4. Integration/Conceptual Model

An integration strategy must be selected to achieve meaningful integration. A joint display is a key component of integration. By merging and comparing the data, the findings could be converged, diverged and expanded. Moreover, a link could be identified between the outcomes (see Supporting file 2). This joint display demonstrates the cross‐validation logic between datasets, showing how meta‐inferences were derived from the integration of quantitative and qualitative results. The qualitative section of the study aimed to uncover the cultural and contextual factors contributing to WPV experiences among interns, which could not be achieved using either a quantitative or qualitative approach in isolation.

The integration highlights the prevalence of WPV and the cultural acceptance of violence in healthcare environments. While quantitative data provided evidence of the prevalence of WPV, the qualitative study revealed a systemic issue in which frequent occurrences of WPV have led interns and staff to accept it as an inevitable part of their jobs (normalisation). This can result from factors including recurrent incidents, insufficient institutional responses, hospital culture, insufficient university preparation and particular factors such as the character of preceptors, the work environment and senior nurses normalising WPV. Hospitals and universities shaped interns’ experiences, with gaps in preparation and support identified in both. An analysis of the quantitative data revealed that almost two‐thirds of the participants received support. The qualitative findings allowed expansion in this understanding by revealing that while support was provided, it was often superficial or inconsistent, lacking sufficient follow‐up. This perception of support highlights how ineffective that support was in reality. Moreover, a significant divergence emerged between the datasets regarding WPV training: while 85% of survey respondents reported receiving training, qualitative interviews revealed widespread dissatisfaction, as interns considered merely reading policy documents to constitute ‘training.’ This discrepancy highlights a critical gap between the official policy and its practical implementation, suggesting that administrative compliance does not equate to meaningful educational preparedness. The data also converged on the issue of underreporting WPV incidents, with one‐third of interns reporting some incidents but not all. In qualitative findings, reporting incidents was associated with fear regarding their career, lack of support, concerns about negative evaluations by their preceptors and fear of ruining their relationships with preceptors and staff nurses. Interns felt unempowered whenever they reported WPV incidents, and the programme organisers took no action. A recurring concept identified across the integration was disempowerment. Many interns had a sense of powerlessness because of their experiences with WPV, the absence of support and the normalisation of violence.

**Figure 4:**
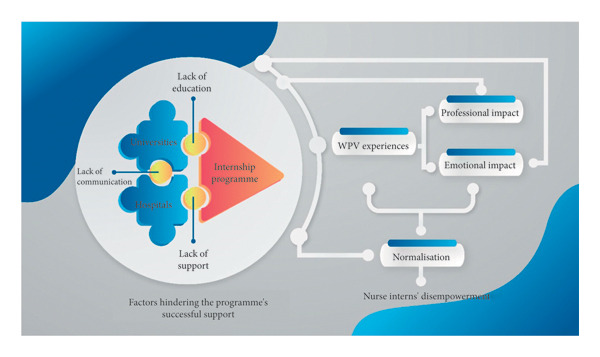
Interns workplace violence and disempowerment conceptual model.

The development of the conceptual model was driven by the need to effectively integrate and interpret the complex data from a mixed‐methods study, offering a new model that enhances understanding of the intertwined factors influencing experiences of WPV and programme support for nurse interns in Saudi Arabia. The model developed for this study (see Figure 4) illustrates the systemic challenges experienced by nurse interns, highlighting educational, support and communication gaps (separate puzzles that reflect these gaps). The two parts of the model, namely, universities and hospitals, are interconnected yet distinct. These entities, in conjunction with the internship programmes (represented by the red triangle), form the foundational structure within which interns operate. As a result, the model critically highlights the disjuncture between these components, symbolised by the gaps between the puzzle pieces. Based on the integrated data analysis, these gaps are identified as systemic deficiencies in education, communication and support—key barriers that hinder the programmes’ provision of effective support. Universities often fail to adequately prepare interns for managing WPV, which is exacerbated by hospitals’ lack of support and mentoring. A lack of communication within internship programmes further exacerbates these issues, leading to significant professional and emotional consequences. The interaction of inadequate education, insufficient support and poor communication amplifies an intern’s professional and emotional consequences. This recurring nature of challenges leads to a normalisation of WPV, leaving nurse interns underpowered and unsupported. The model calls for systemic reforms to address these gaps and ensure the success and well‐being of nurse interns.

To contextualise and enhance the model’s insights, and to appreciate how it may be applied in practice, existing theories are re‐visited, particularly the socio‐ecological model [48], transition shock theory [49], and structural [50] and psychological empowerment [51]. The conceptual model utilises a socio‐ecological lens to explicitly integrate the shared responsibility of both universities and hospitals in the intern transition process. This dual‐organisational focus provides a more comprehensive understanding of WPV and enables the development of more effective, multi‐level interventions. The model expands transition shock theory by incorporating the dual emotional and professional impacts of WPV, highlighting how navigating violent environments intensifies the challenges of role adaptation. It integrates psychological and structural empowerment theories to demonstrate how organisational failures—from both universities and hospitals—normalise violence and lead to intern disempowerment. A key contribution lies in framing normalisation and disempowerment as critical outcomes, clarifying how institutional shortcomings shape intern experiences and responses.

## 5. Discussion

This research has examined interns’ experiences of WPV in Saudi Arabia throughout the nursing internship programmes. Interns highlighted many violent behaviours, including physical actions (i.e., punching) and verbal abuse (i.e., threats). A high number of interns (90%, *n* = 123) reported witnessing or being subjected to WPV throughout the programmes. Other studies’ findings support this in Arabian [52, 53] and Asian [54] countries. The primary sources of WPV were identified as patients and their relatives. Moreover, physical violence was experienced more by male interns than female interns, and this could be because male interns are prohibited from treating female patients in Saudi Arabia. Thus, caring solely for male patients may render male interns more vulnerable to WPV. On the other hand, male interns are often expected to handle physical aggression. This is perhaps because society has aligned physical strength and authority with masculinity.

Similar frequencies of WPV have been identified in global studies [17, 31]. This study focuses specifically on hospital wards (i.e., emergency, surgical, medical, and intensive care), which may cause higher WPV rates. Differences in prevalence between studies could be attributed to different time frames and reporting systems. It is difficult to compare the findings of this study with those of other relevant articles because this is the first study of its kind to examine this topic and because any differences could be attributed to different time frames (i.e., examining the topic during the internship programmes). Meanwhile, other studies [55, 56] examined NGN experiences after completing the internship programmes. In turn, this can generate varying results. Furthermore, we highlighted a crucial distinction: while the high prevalence aligns with global trends, the unique vulnerability of the intern’s role within the Saudi cultural and hierarchical structure represents a significant divergence from studies on NGNs or students, making direct prevalence comparisons less insightful than comparing the systemic factors that enable it.

In contrast to other existing studies [57, 58], this study did not observe high turnover rates among interns, and this may be because the completion of the 1‐year programme is mandatory under Saudi regulations. Nonetheless, the emotional impact of WPV on interns was significant. Participants stated that sufficient support can increase the resilience of interns and their ability to overcome challenges. Due to a lack of support, there is a persistent cycle of transition shock and vulnerability to WPV, leading interns to normalise such behaviours. This, in turn, results in disempowerment.

The results of this research are in alignment with previous studies, where these programmes have proved their effectiveness in easing interns’ transition [59], supporting interns, improving their clinical skills [60], enhancing their resilience [61] and ultimately enhancing their retention [29]. Nonetheless, although the programmes have many advantages in terms of helping graduate nurses transition to their new clinical roles, this study revealed that the current programmes did not provide the support required by interns who experience WPV.

This study created a WPV and disempowerment model to generate an in‐depth, comprehensive understanding of the factors impacting interns’ WPV experiences and hindering the programmes’ provision of support. Each model component has been contextualised within existing literature, theories and research questions and discussed below.

### 5.1. Education

This study found that younger interns may be more likely to experience WPV, especially in high‐stress environments like emergency departments. In addition, interns who progress beyond the first 4 months of the programmes are significantly more likely to become violent, suggesting that orientation and lectures may not prepare them for the practical challenges of clinical practice. Increasing risk is consistent with the concept of ‘transition shock’ ([49], p. 13), in which new nurses struggle to shift from theoretical learning to patient care. The importance of education about WPV for interns is highlighted in this model. Many interns experience anxiety and stress, which is further exacerbated when educational institutions and healthcare institutions insufficiently prepare them. Issues, including supervision obligations, insufficient time and patient loads, aggravate the stress experienced during the transitional period [62]. Furthermore, poor training about WPV leaves interns vulnerable, underscoring the importance of education during the nursing curriculum. Because of the current lack of education in this area, it is hard for interns to develop a professional identity and remain committed to patient care as they experience violence, burnout and dissatisfaction with their work [63]. Therefore, interns should receive comprehensive WPV education during their studies and internships [64, 65]. Important educational components include training programmes that cover cognitive rehearsal [66], role‐play activities [67], simulations [68, 69] and online courses [70]. After education sessions related to WPV, there was a significant increase in knowledge [68, 71]. It is imperative that the programmes involve university–hospital collaboration and combine WPV modules, such as recognition, de‐escalation, reporting, and self‐care. Moreover, it should promote communication and support throughout the placement.

### 5.2. Communication

The second critical factor covered in the model is the need for more communication between universities and hospitals relating to WPV for interns. Bronfenbrenner’s [48] socio‐ecological model highlights organisational‐level communication problems’ impacts on interns’ experiences, causing interpersonal conflicts and knowledge gaps. The misalignment between the priorities of universities and hospitals throughout the transition period causes a major knowledge gap [28, 72]. This research has also uncovered a disparity between universities and hospitals regarding preparation approaches, making the transition period difficult for interns. The programmes sometimes operate with hardly any collaboration, which prevents effective communication. This also impacts preceptors, causing misunderstandings and poor treatment of interns. This can cause professional identity confusion, as preceptors treat interns as students instead of nurses. The lack of clear guidance and communication between programme organisers and preceptors exacerbates this problem. A number of researchers have found that a smooth transition is promoted through effective communication between universities and hospitals, which reduces disempowerment [73, 74]. Even though the latter study did not examine the opinions of preceptors, two qualitative phenomenological studies have examined the experiences of registered nurses regarding nursing students [28, 75], with the findings highlighting a need to establish a clearly defined role for nursing students in clinical practice. In addition, the need for formal collaboration, enhanced communication and preceptorship programmes has been highlighted in this work to address issues of confusion regarding professional identity and the gap between academia and practice. There is an evident need for comprehensive support to strengthen interns’ resilience throughout the transition process.

### 5.3. Support

The model highlights the importance of support for interns with WPV. Poor and inconsistent support from universities, hospitals and programme coordinators has increased the risk of WPV for interns. Interns primarily turn to preceptors, ward managers and programme organisers for emotional support but have limited (if any) access to counselling or peer support. More comprehensive support is needed to help interns manage WPV incidents.

Professional support from mentors and experienced preceptors is critical in ensuring the successful professional development of interns throughout the transition period. Interns can be empowered to cope with WPV incidents when preceptors share their experiences and advocate for them. On the other hand, not all preceptors are supportive, and some instigate WPV acts themselves. Several factors can cause unsupportive preceptor behaviour, such as the intricate healthcare environment, workload pressures and concerns about being replaced in the Saudi workforce (Saudisation). Meanwhile, staff conflicts and an unsupportive environment increase the risk of horizontal and oppressive behaviour [76, 77].

This study uses the phrase ‘nurses eat their young’ to highlight the hostile culture in healthcare settings ([78], p. 10) and the problems interns encounter as they navigate their relationships with experienced staff. Workload, burnout and stress increased significantly during the pandemic, adversely influencing preceptors; ability to guide their interns [79]. This study’s findings highlight this culture’s impacts on interns, attributing such issues to the Saudi culture.

Furthermore, a key objective of Saudi Vision 2030 is to train and recruit more Saudi nurses (Saudisation is defined as replacing the non‐Saudi workforce in Saudi Arabia with a national workforce with the required skills and qualifications), and the MOH started implementing this policy within hospitals, which meant replacing non‐Saudi staff nurses with Saudi nurses [6, 80]. These dynamics are increasing the emotional challenges experienced by non‐Saudi preceptors. Freire’s [81] oppressed group theory explains how oppressed groups internalise the values of dominant groups, causing internal conflicts, which has been introduced to nursing. Moreover, the turnover rate among interns is low, in spite of the emotional effects of WPV, primarily because of the mandatory nature of completing the internship programmes in Saudi Arabia. Nonetheless, there is an urgent need to provide interns with professional, emotional, educational and organisational support throughout the transition period. Without this support, the focus will continue to be solely on improving clinical skills without concern for interns’ well‐being and needs. Developing an empowered, resilient nursing team is vital in ensuring interns’ well‐being and high‐quality patient care.

### 5.4. Normalisation of WPV and Interns’ Disempowerment

The model incorporates WPV normalisation and the consequential disempowerment it caused interns. Given their limited knowledge and lack of support, they are more likely to downplay or even normalise WPV actions at work, which can lead to violence being tolerated. In turn, this can generate a hostile work environment. As supported by the findings of other relevant studies, WPV can have significant effects on the emotions [17] and professional [82] lives of interns, including anxiety, depression, fear, isolation [10], work dissatisfaction and confusion over their professional identity [83].

Furthermore, a fragmented programmes approach is emerging because of the poor communication between universities, hospitals and programme coordinators, which is hindering interns from accessing the education and support they need. This can result in graduate nurses normalising WPV as a routine part of their job. Frustration and anger can ultimately cause WPV. Studies have shown that psychological empowerment plays a critical role in preventing WPV, and such incidents can be reduced by developing interventions that increase empowerment [84]. In addition, insufficient training and support from programme coordinators compromise interns’ psychological empowerment [51], rendering them powerless and frustrated. There is a significant relationship between structural and psychological empowerment and a positive relationship between increased empowerment, work engagement and burnout prevention [85]. The study thus highlights the importance of structural and psychological empowerment in preventing interns from experiencing WPV.

Furthermore, the cultural context is important since interns’ perceptions of WPV are influenced by Saudi Arabian cultural standards, which emphasise deference to authority, upholding social harmony and respecting hierarchy, meaning that interns often tolerate WPV incidents to avoid confrontation. Cultural inclinations to avoid conflict, fear of stigmatisation and professional anxiety have been identified as key contributors to the underreporting of WPV incidents. Almutairi [86] claim that traditional values impact nurses’ reactions to WPV. The conservative culture of Saudi Arabia likely influences this, with interns fearing that the healthcare institution’s reputation would be tarnished. Furthermore, Alsaleem et al. [87] explain that sexual harassment in healthcare settings has been poorly investigated in the Middle East and that female healthcare workers are hesitant to address the issue because of cultural sensitivities and stigmatisation from their families and the wider community. This calls for culturally sensitive means of encouraging disclosure, minimising the associated stigma and providing interns with targeted support mechanisms to help them deal with WPV.

Avolio et al. ([88], p. 242) describe authentic leadership as a ‘pattern of transparent and ethical leadership behaviour that promotes openness and information sharing whilst allowing followers to make an input’. This is critical in addressing WPV, as leaders play a critical role in developing a positive workplace culture. A relationship has been identified by Laschinger and Read [89] between authentic leadership and reduced bullying among new nursing graduates. Nonetheless, further research is required to examine the association between authentic leadership, WPV exposure and support for interns. According to the model employed in this study, insufficient support, education and communication adversely impact interns’ experiences of disempowerment and WPV. Moreover, Monje‐Amor et al. [85] support this model, highlighting that organisational and professional factors significantly impact interns’ confidence and feelings of empowerment. The research also indicates that including more practical activity hours in the nursing curriculum, enhancing work conditions, developing orientation programmes and establishing a positive reputation in nursing are critical in improving interns’ empowerment and reducing WPV.

### 5.5. Implications

This study on WPV experienced by nurse interns in Saudi Arabia reveals significant implications for nursing education, practice and policy. It highlights the need to explicitly address WPV in nursing internship programmes, exposing gaps in communication and support between universities and hospitals. The findings challenge existing theoretical models and offer a novel conceptual framework for programme organisers. Practically, the study underscores deficiencies in curricula and clinical guidelines that leave interns vulnerable, potentially affecting their well‐being and patient care quality. It also reveals a disconnect between recent policy changes and interns’ awareness, suggesting a need for better integrating WPV policies into nursing education. The study emphasises the importance of creating supportive work environments and culturally sensitive approaches to addressing WPV in Saudi Arabia’s conservative culture. These findings call for collaborative efforts among educational institutions, healthcare organisations and policymakers to improve intern experiences and create safer healthcare environments.

### 5.6. Recommendations

• Review and update nursing education curricula to include WPV prevention, recognition and response, incorporating case studies, practical scenarios and simulation training and to align university education with WPV realities in healthcare settings.
• Promote authentic leadership among programme organisers to improve support systems and encourage WPV reporting.
• Establish mentorship programmes pairing experienced nurses with interns and provide ongoing professional development in resilience, communication and coping skills.
• Enhance collaboration between universities, hospitals and programme organisers to improve intern transition and reduce WPV vulnerability.
• Advocate for policy revisions to explicitly address WPV in healthcare organisations.
• Incorporate violence prevention strategies into nursing education and internship programmes.
• Confidential counselling services should be established within hospitals through MOH funding, supported by awareness *programs to integrate mental healthcare into* professional development.
• Provide training on the MOH reporting system (including a support telephone line (937) for reporting any acts of WPV operating in parallel to police channels and the new penalties to control and prevent WPV). However, considering the cultural sensitivity of Saudi Arabia, this system needs to have a high level of confidentiality.
• Awareness campaigns should be created in the healthcare community and among the general public to shed light on WPV, especially among NGNs and interns.
• Promote a respectful work environment, including proper introduction of interns to patients.
• In order to determine the effectiveness of these interventions, longitudinal studies should be conducted to assess interns’ experiences with WPV, their transition to practice and their long‐term career outcomes.

### 5.7. Research Strengths and Limitations

The strengths of this research are numerous. For example, the mixed‐method design facilitates an in‐depth understanding of interns’ experiences by overcoming the limitations of each method. Several actionable recommendations have been made following the proposed model, which can be used to create new policies and interventions that promote a more supportive work environment during the internship placement. Nonetheless, this study also has a number of limitations. For example, using a cross‐sectional survey brings about potential sampling bias, which can influence participants’ responses. Moreover, the transferability of the qualitative findings is limited by the small sample size, use of Zoom for interviews and lack of nonverbal cues. The qualitative investigations also have temporal constraints, meaning that differences in interns’ experiences over time are not considered. The uneven geographical distribution of the sample (Riyadh: 62.6%, Jeddah: 37.4%) may limit the generalisability of findings across all regions. However, qualitative analysis revealed no substantive differences in core themes between the two cohorts. *Finally, very few studies have examined verbal abuse experienced by Saudi nurses throughout their internships. Thus, it is difficult to compare and review relevant literature.*

## 6. Conclusion

Interns are exposed to various sources of WPV during the transition to clinical practice, and, at present, they are not provided with sufficient support. To ensure the success of internships, universities must work closely with hospitals to support and empower interns by addressing their unique needs. A critical evaluation should be performed on the model and the programmes, and the factors impeding programmes support should be carefully examined.

## Disclosure

Khadijah Alshawush, Nutmeg Hallett and Caroline Bradbury‐Jones approved the manuscript version to be published.

## Conflicts of Interest

The authors declare no conflicts of interest.

## Author Contributions

Khadijah Alshawush: conceptualisation and design of the study. Khadijah Alshawush: data collection, analysis and interpretation. Nutmeg Hallett and Caroline Bradbury‐Jones: reviewed the analysis. Khadijah Alshawush: drafting and writing the manuscript. Nutmeg Hallett and Caroline Bradbury‐Jones: revising and editing the manuscript critically for important intellectual content.

## Funding

This research received no specific grant from any funding agency in the public, commercial or not‐for‐profit sectors.

## Supporting Information

Additional supporting information can be found online in the Supporting Information section.

## Supporting information


**Supporting Information 1** Supporting File 1: The good reporting of a mixed methods study (GRAMMS) checklist.

**Supporting Information 2** Supporting File 2: Mixed‐method joint display integration table.

## Data Availability

The data supporting the findings of this study are not publicly available because of the sensitive nature of the research topic and because they form part of a doctoral thesis. Deidentified data may, however, be made available from the corresponding author upon reasonable request and subject to appropriate conditions.
