# Correction to “Nurse Interns’ Experiences of Workplace Violence During Internship Programme Enrolment: A Convergent Mixed‐Method Study”

**DOI:** 10.1155/jonm/9828323

**Published:** 2026-08-04

K. Alshawush, N. Hallett, and C. Bradbury‐Jones, “Nurse Interns’ Experiences of Workplace Violence During Internship Programme Enrolment: A Convergent Mixed‐Method Study,” *Journal of Nursing Management*, 2025, 7421931, 18 pages, 2025, https://doi.org/10.1155/jonm/7421931.

In the originally published version of this article, the affiliation for the first author, Khadijah Alshawush, did not conform to the official institutional format.

The correct affiliation is as follows:

Public Health Department, Faculty of Nursing, King Abdulaziz University, Jeddah, Saudi Arabia.

The online version of this article has been corrected accordingly.

We apologize for this error.

