# Supplementary material for: Nurse Interns’ Experiences of Workplace Violence During Internship Programme Enrolment: A Convergent Mixed‐Method Study
Source: J Nurs Manag. 2025 Oct 7;2025:7421931. doi: 10.1155/jonm/7421931 (PMC12520816; doi:10.1155/jonm/7421931)
Supplement: Supplementary file 1 — Supporting Information 1 Supporting File 1: The good reporting of a mixed methods study (GRAMMS) checklist. [file JONM-2025-7421931-s002.docx]

Supplementary Table 1: The Good Reporting of a Mixed Methods Study (GRAMMS) Checklist

| **Guideline** | **Section: Page** |
| --- | --- |
| Justification to use a mixed methods approach to the research  question | Method: p. 4 |
| Articulation of the design in terms of purpose, priority, and sequence of methods | Design: p. 4 |
| Describe each method in terms of sampling, data collection and analysis | Data collection: pp. 4-5  Data Analysis: p. 6 |
| Delineate where and how integration occurs and who has participated in it | Joint display: pp. 13- 14 |
| Describe any limitation of one method associated with the  presence of another | Strength and limitations: p. 20 |
| Describe insights gained from mixing or integrating methods | Joint display: pp. 13-14  Discussion: pp. 15-18 |
