# Supplementary material for: Nurse Interns’ Experiences of Workplace Violence During Internship Programme Enrolment: A Convergent Mixed‐Method Study
Source: J Nurs Manag. 2025 Oct 7;2025:7421931. doi: 10.1155/jonm/7421931 (PMC12520816; doi:10.1155/jonm/7421931)
Supplement: Supplementary file 2 — Supporting Information 2 Supporting File 2: Mixed‐method joint display integration table. [file JONM-2025-7421931-s001.docx]

Supplementary Table 2: Mixed-method joint display integration

| **Main integrated section** | **Aspect/themes/sub-themes** | **Quantitative findings (percentages)** | **Qualitative findings** | **Meta-inferences** | **Converged/diverged/ expanded** |
| --- | --- | --- | --- | --- | --- |
| Experiences of WPV | Experiences of WPV/ nurse interns’ vulnerability | Most interns (92.2%) had experienced WPV | Experiences of WPV  Most interns reported having experienced different acts of WPV (physical and verbal)  Interns generally normalised unsafe working conditions  *It is the norm to be treated like that*. (Participant 20) | Interns experience different acts of WPV, and they are vulnerable to it  As a result of having experienced WPV and not being supported during this experience, interns accept being victims of WPV | **Converged/expanded** Quantitative findings expanded the qualitative results and provided details on the different acts of WPV and their frequency, and qualitative findings provided reasons for normalising it |
| Perceptions of support | Advantages of the programme | Forty eight percent (n = 48) of interns found that the management during the programme was supportive and tried to find solutions after a violent attack, and provided relief to enable the victim to leave after the incident | A few interns felt supported during the programmes; however, those interns had not reported WPV  Interns felt more confident in working in environments with WPV; they were satisfied with the clinical training that the programmes provided to them, and felt that they improved their clinical and communication skills | The programmes provided emotional support to interns during their experience of WPV. The programmes were also successful in improving interns’ resilience in dealing with such experiences and easing their transition to clinical practice | **Converged/Expanded** Qualitative findings expanded the qualitative results and provided more advantages of the programmes |
| Factors hindering support | Factors impeding the programmes’ provision of support | The majority of interns (n = 88, 71.5%) believed that their managers were not very concerned about their safety at work. Moreover, some interns (n = 45, 45%) were not given any relief from work after WPV incidents | University factors  There was inadequate preparation for nursing students in terms of dealing with WPV incidents and no follow-up for the interns.  Hospital factors  Most interns experienced several issues within the work environment, such as work overload and ignorance from their preceptors and senior staff. Interns believed that expatriate preceptors were threatened by new nursing graduates and felt that they were replaceable | Lack of communication between the programme organisers, along with insufficient support and education, influenced the support provided by the programmes and contributed to WPV experiences | **Expanded**  Qualitative findings provided details about factors that could hinder the programmes’ support |
|  | Availability of training and education | The majority of interns (n = 105, 85.4%) agreed about the availability of training regarding WPV prevention. However, most of that training entailed enrolling in self-defence courses independently and/or reading hospital policies | All interns reported that there were no education sessions or training regarding dealing with and preventing WPV | Although interns reported receiving training regarding WPV and their legal rights, this training involved mainly reading hospital policy, and it was not sufficient, according to interns who had experienced WPV | **Diverged**  Interns in the quantitative study indicated that they had received training; in contrast, those in the interviews reported that they had not received specific training about WPV, and provided details about what they meant by training |
| Impact of WPV | The impact of WPV and insufficient programme support for interns | Interns experienced difficulties concentrating on their work after WPV incidents (26.1%) and working in the same environment (15.3%). They also had psychological symptoms, such as fear (9%), and physical symptoms, such as headaches (9%) | Emotional impact  Interns were emotionally affected and reported feeling depressed, stressed, fearful and helpless  Professional impact  WPV impacted interns’ job performance, and they expressed an intention to leave the programmes and not to work in the same hospitals. Moreover, some identity confusion arose about interns’ role (whether they were students or new graduates) | Interns experienced emotional and professional consequences as a result of WPV experiences and the insufficient support provided by their programmes | **Converged/Expanded** Qualitative findings expanded on the professional impact of WPV |
|  | Underreporting of WPV incidents | Most of the interns (69.1%) knew their legal rights and where and how to report WPV incidents. However, only half of them actually reported these incidents | Interns reported that they did not report violent incidents due to the lack of support and fear of professional repercussions  *Because she [the preceptor] evaluates me at the end of the programme, I did not report it.* (Participant 2) | There was a dilemma about reporting incidents of WPV among interns as a result of insufficient support and fear of professional consequences | **Converged/ Expanded** Qualitative findings provided reasons for the underreporting of WPV incidents |
|  | Interns felt disempowered | Almost half of the interns felt that they had no control over their safety in their workplace (47.2%) | Most of the interns felt powerless to make any changes in terms of dealing with and preventing WPV | A lack of WPV education and support from the programmes, along with ineffective communication between the programme organisers and interns, left interns feeling disempowered | **Converged/Expanded** Qualitative findings provided potential reasons for interns’ disempowerment |
| Needs of interns | Interns’ needs and suggestions | Nearly all interns (n = 118, 95.9%) believed that improvements are required to help improve working conditions in relation to WPV during the internship period. Training on how to prevent WPV was the most frequently suggested solution (58.5%) | Needs Interns mainly needed education, support, effective communication and empowerment  Suggestion Interns believed that education sessions about dealing with WPV and prevention, adequate support, effective communication, and continued evaluation of the programmes could help to improve the programmes and empower them | Interns suggested some solutions that could improve their working conditions. These solutions were in line with their needs | **Converged/Expanded** Interns provided more specific needs and solutions during interviews |
